# WZB117-Induced Glucose Hypometabolism Triggers Mitochondrial Dysfunction and Amyloidogenic Processing in an In Vitro Primary Forebrain Neuron Model

**DOI:** 10.7759/cureus.106239

**Published:** 2026-03-31

**Authors:** Sajidali S Saiyad, Jay Prakash S Rajput, Sandeep M Horo, Komal Kumari, Prasanta Chatterjee Biswas, Gnanadesigan Ekambaram, Anju Chouhan, Surbhi Ranga

**Affiliations:** 1 Physiology, Pacific Medical College and Hospital, Pacific Medical University, Udaipur, IND; 2 Physiology, Shri Gorakshnath Medical College, Hospital and Research Centre, Gorakhpur, IND; 3 Medicine, Netaji Subhas Medical College and Hospital (NSMCH) Jamshedpur, Jharkhand, IND; 4 Biochemistry, Maharishi Markandeshwar Institute of Medical Sciences and Research, Maharishi Markandeshwar (Deemed to be University), Ambala, IND; 5 Centre for Distance and Online Education (CDOE), Parul University, Vadodara, IND; 6 Physiology, Nootan Medical College and Research Centre, Sankalchand Patel University, Visnagar, IND; 7 Physiology, Dr. Sampurnanand Medical College, Jodhpur, IND; 8 Physiology, Ananya College of Medicine and Research, Ahmedabad, IND

**Keywords:** alzheimer’s disease, amyloidogenic processing, cerebral glucose hypometabolism, metabolic neurodegeneration, mitochondrial dysfunction, neuronal energy metabolism, β-secretase (bace1)

## Abstract

Background

Alzheimer’s disease (AD) is a progressive neurodegenerative disorder in which cerebral glucose hypometabolism represents one of the earliest and most consistent pathological abnormalities, often preceding classical amyloid and tau pathology. Despite strong clinical evidence, the causal contribution of impaired neuronal glucose utilization to AD-related cellular alterations remains incompletely understood.

Objective

This study aimed to determine whether selective inhibition of neuronal glucose transport induces AD-relevant cellular stress responses, including mitochondrial dysfunction and amyloidogenic processing, in a controlled primary neuronal system.

Methods

Primary forebrain neurons were established from embryonic chick brain and subjected to glucose hypometabolism using WZB117, a pharmacological inhibitor of facilitative glucose transporters. Neuronal viability and cytotoxicity were assessed by trypan blue exclusion and lactate dehydrogenase release assays. Mitochondrial membrane potential was evaluated using TMRE (tetramethylrhodamine ethyl ester) fluorescence, while amyloidogenic processing was quantified by measuring β-secretase (BACE1) activity and intracellular Aβ42 levels. β-hydroxybutyrate was employed to assess metabolic rescue.

Results

Inhibition of glucose transport resulted in a significant increase in neuronal cell death and cytotoxicity, accompanied by a pronounced reduction in mitochondrial membrane potential, indicating severe bioenergetic failure. Concurrently, amyloidogenic processing was markedly enhanced, as evidenced by elevated BACE1 activity and increased intracellular Aβ42 accumulation. Metabolic supplementation with β-hydroxybutyrate significantly attenuated neuronal death, restored mitochondrial function, and suppressed amyloidogenic alterations.

Conclusions

These findings demonstrate that acute neuronal glucose hypometabolism induces mitochondrial depolarization and increased amyloidogenic signaling in primary neurons, suggesting that metabolic stress may contribute to cellular processes associated with AD. The study identifies impaired neuronal bioenergetics as a potential upstream contributor to AD-related cellular pathology and supports further investigation of metabolism-focused strategies in AD research.

## Introduction

Alzheimer’s disease (AD) is the most prevalent neurodegenerative disorder and the leading cause of dementia worldwide, accounting for approximately 60%-70% of all dementia cases and affecting over 55 million individuals globally, with projections expected to exceed 130 million by 2050 [1,2]. Current therapeutic strategies are predominantly symptomatic and provide limited disease-modifying efficacy.

Neuropathologically, AD is characterized by the accumulation of extracellular amyloid-β (Aβ) plaques and intracellular neurofibrillary tangles composed of hyperphosphorylated tau protein, accompanied by synaptic dysfunction, neuronal loss, and progressive cognitive decline [3-5]. The amyloid cascade hypothesis proposes that aberrant production and aggregation of Aβ, particularly the Aβ₁₋₄₂ species, initiates a sequence of downstream events including tau pathology, oxidative stress, neuroinflammation, and neurodegeneration [6]. Complementing this view, tau-centered models emphasize microtubule destabilization and impaired axonal transport as critical mediators of neuronal dysfunction [7]. However, repeated clinical failures of amyloid- or tau-targeted therapies suggest that these hallmark lesions may not fully explain disease initiation and progression, especially in sporadic late-onset AD.

One of the most consistent and early abnormalities observed in AD is cerebral glucose hypometabolism. Functional neuroimaging studies using ¹⁸F-fluorodeoxyglucose positron emission tomography (FDG-PET) have demonstrated region-specific reductions in glucose utilization in the temporoparietal cortex, posterior cingulate, and hippocampus of AD patients [8,9]. Importantly, such metabolic deficits are detectable in individuals with mild cognitive impairment and even in asymptomatic subjects at genetic risk, often preceding overt amyloid deposition or clinical symptoms [10]. These findings strongly suggest that impaired brain energy metabolism is not merely a consequence of neurodegeneration but may represent an early and causative pathogenic event.

At the cellular and molecular levels, reduced cerebral glucose metabolism in AD is closely linked to dysfunction of glucose transport and insulin signaling pathways. Neurons are highly energy-dependent cells that rely primarily on facilitated glucose transport mediated by glucose transporter isoforms such as GLUT1 and GLUT3 [11]. Postmortem studies and experimental models have demonstrated reduced expression of glucose transporters at the blood-brain barrier and within cortical neurons in AD brains, correlating with disease severity and tau pathology [12,13]. Furthermore, impaired glucose availability has been shown to decrease O-GlcNAcylation of tau, thereby promoting tau hyperphosphorylation and cytoskeletal instability [14]. These observations have led to the conceptualization of sporadic AD as a form of “type 3 diabetes,” characterized by brain-specific insulin resistance and metabolic failure [15,16].

Despite strong clinical and mechanistic evidence linking glucose hypometabolism to AD, direct experimental interrogation of this pathway remains limited by model constraints. In vivo mammalian models, while invaluable, are costly, ethically demanding, and often confounded by systemic metabolic effects that obscure neuron-specific mechanisms [17]. Conventional in vitro neuronal cultures lack architectural organization and metabolic complexity, limiting their translational relevance. Consequently, there is a critical need for alternative experimental systems that allow controlled manipulation of neuronal glucose metabolism while preserving key cellular and structural features of the nervous system.

The embryonic chick brain represents a robust yet underutilized model for neurobiological research. Chick embryos offer several advantages, including low cost, year-round availability, rapid development, and fewer ethical constraints, compared with mammalian models [18]. Primary cultures derived from chick forebrain neurons exhibit well-defined axonal and dendritic morphology, predictable growth patterns, and neurochemical properties comparable to mammalian central neurons [19,20]. These features have enabled their successful use in studies of neurite outgrowth, synaptic development, and neuronal metabolism, supporting their suitability for mechanistic investigations of neurodegenerative processes.

However, the potential of chick forebrain primary neuronal cultures to model glucose hypometabolism-induced Alzheimer’s-like pathology has not been systematically explored. In particular, pharmacological inhibition of neuronal glucose transport provides a direct means to induce energy deficiency and examine downstream pathogenic cascades independent of systemic metabolic influences. Such an approach offers a unique opportunity to dissect the causal role of neuronal glucose hypometabolism in AD-relevant cellular alterations. A controlled primary neuronal system refers to an experimental in vitro model in which neuronal cells are studied in isolation under defined environmental and metabolic conditions, allowing precise manipulation of specific variables such as glucose availability without interference from systemic factors, including vascular, hormonal, or inflammatory influences. Such systems are particularly valuable for dissecting cell-autonomous mechanisms underlying neurodegenerative processes, including metabolic stress responses relevant to AD.

Objective and novelty of the present study

The present study aimed to determine whether selective impairment of neuronal glucose utilization induces cellular stress responses relevant to AD, including mitochondrial dysfunction and altered amyloid precursor protein processing, in a controlled primary neuronal system.

While cerebral glucose hypometabolism is well documented in clinical and in vivo studies of AD, direct experimental evidence linking isolated neuronal glucose transport inhibition to amyloidogenic processing and mitochondrial dysfunction remains limited.

To our knowledge, this is the first study to systematically demonstrate that pharmacological inhibition of neuronal glucose transport using WZB117 in primary chick forebrain neurons is sufficient to induce coordinated bioenergetic failure and amyloidogenic alterations in a controlled in vitro system, independent of systemic metabolic influences.

Furthermore, the study integrates dose-response, time-course, and metabolic rescue approaches, providing mechanistic insight into the causal role of neuronal energy deficiency in AD-related cellular pathology. These findings extend existing models by establishing a simplified yet biologically relevant platform for investigating metabolic contributions to neurodegeneration.

## Materials and methods

Study design and experimental overview

This in vitro experimental study was designed to investigate the cellular consequences of neuronal glucose hypometabolism relevant to AD pathology using primary forebrain neuronal cultures derived from chick embryos. Glucose hypometabolism was induced pharmacologically using the glucose transporter inhibitor WZB117 to directly impair neuronal glucose utilization in a controlled cellular environment. The experimental approach allowed mechanistic assessment of neuronal viability, mitochondrial membrane potential, and amyloidogenic processing independent of systemic metabolic confounders. All experiments were performed under standardized culture conditions with parallel controls to ensure reproducibility.

Ethical approval

All experimental procedures involving chick embryos were reviewed and approved by the Institutional Ethical Committee (IEC) of Maharishi Markandeshwar Institute of Medical Sciences and Research (MMIMSR), Mullana, Ambala, India (Project No.: IEC/2182). The project entitled “Developing a toxin-based model of neurodegeneration relevant to AD using primary neuronal culture from chicken brain” was approved on March 15, 2022, and recommended for a period of two years. Chick embryos were used before hatching in accordance with institutional and national ethical guidelines, and all efforts were made to minimize biological material usage and experimental variability. All experiments were conducted between March 2022 and February 2024, within the approved project period.

Materials and reagents

All chemicals and reagents used were of analytical grade. Neurobasal medium, fetal bovine serum, penicillin-streptomycin, WZB117, tetramethylrhodamine ethyl ester (TMRE), tris-EDTA buffer, and bicinchoninic acid (BCA) protein assay reagents were obtained from Merck (India). Trypan blue, nicotinamide adenine dinucleotide (NADH), dimethylsulfoxide (DMSO), trypsin, glycerol, ethanol, and methanol were procured from HiMedia (Mumbai, India). Standard laboratory glassware was sourced from Borosil (India), and sterile plasticware, including culture plates and flasks, was obtained from Genaxy (New Delhi, India).

Chick embryos and incubation conditions

Fertilized White Leghorn (Gallus gallus domesticus) eggs were procured from a certified commercial supplier and incubated in a humidified egg incubator at 37.5°C-38.5°C with automatic rotation. Embryos were staged according to standard developmental criteria, and embryonic day 14 (E14) embryos were selected for neuronal isolation. Eggs were surface-sterilized with 70% ethanol before dissection to maintain aseptic conditions.

Isolation and culture of primary forebrain neurons

Primary neuronal cultures were prepared using established protocols with minor modifications [18,19]. Eggs were opened at the blunt end, and embryos were carefully extracted and transferred to sterile Petri dishes containing Dulbecco’s balanced salt solution. Forebrains were dissected under sterile conditions and mechanically minced. Tissue fragments were washed repeatedly with phosphate-buffered saline containing calcium and magnesium to remove blood and yolk residues.

Enzymatic dissociation was performed using 0.25% trypsin combined with versene, prewarmed to 37°C, for 15-20 minutes with gentle agitation. Enzymatic activity was terminated by the addition of Eagle’s minimum essential medium. The resulting cell suspension was centrifuged at ambient temperature for 10 minutes, and the cell pellet was resuspended in fresh medium. Cells were counted, diluted appropriately, and seeded onto culture plates. Cultures were maintained in a humidified CO₂ incubator and allowed to stabilize before experimental treatments.

Induction of glucose hypometabolism

To induce neuronal glucose hypometabolism, stabilized forebrain neuronal cultures were treated with WZB117, a pharmacological inhibitor of facilitative glucose transporters, for 48 hours. Control cultures received vehicle treatment alone. All experimental conditions were performed in parallel with independent biological replicates. To confirm target engagement and rule out nonspecific toxicity, glucose uptake was measured in parallel cultures using the fluorescent glucose analog 2-NBDG (2-(N-(7-nitrobenz-2-oxa-1,3-diazol-4-yl)amino)-2-deoxyglucose). Following 24 h treatment with WZB117 (60 µM), neurons were incubated with 100 µM 2-NBDG for 30 minutes at 37°C and washed, and then fluorescence was measured at λex 465 nm/λem 540 nm. Uptake was normalized to protein content and expressed as a percentage of the control. Additionally, to distinguish between effects of glucose deprivation versus direct compound toxicity, parallel cultures were treated with WZB117 in medium containing equimolar concentrations of the non-metabolizable glucose analog 3-O-methyl-D-glucose (60 mM) in place of glucose. To establish concentration-dependent effects, preliminary experiments were conducted with WZB117 at concentrations of 0, 10, 30, 60, and 100 µM for 48 h, assessing cell viability (trypan blue exclusion) and BACE1 activity. Based on these results, 60 µM was selected as the minimum concentration producing maximal effects on both endpoints and was used for all subsequent experiments. For time-course analysis, separate cultures were treated with 60 µM WZB117 for 12, 24, 48, and 72 h, with viability and mitochondrial membrane potential assessed at each time point.

Based on preliminary dose-response experiments (0-100 µM) and time-course analysis (12-72 h), 60 µM WZB117 for 48 hours was selected as the optimal condition for all primary outcome assessments.

Sample size determination

Sample size (n = 5 independent cultures per group) was selected based on prior studies using similar primary neuronal culture systems and endpoints, in which n = 4-6 per group was sufficient to detect statistically significant differences with acceptable variability [8,9,13,14]. This sample size was consistent with established reproducibility standards for in vitro primary neuronal culture studies and was maintained across all experiments to ensure balanced statistical comparisons. This replicate size was consistent with accepted practice in mechanistic AD research and was sufficient to detect treatment-related differences with appropriate statistical confidence.

Assessment of cell viability and cytotoxicity

Neuronal viability was assessed using the trypan blue exclusion assay. Following treatment, cells were harvested, stained with trypan blue, and quantified using an automated cell counter. Live and dead cells were expressed as a percentage of the total cell count relative to untreated controls.

Cytotoxicity was further evaluated by measuring lactate dehydrogenase (LDH) release into the culture medium. LDH activity was determined spectrophotometrically by monitoring NADH oxidation at 340 nm using pyruvate as substrate. Enzyme activity was normalized to total protein content and expressed as a percentage of maximal LDH release obtained from cells treated with 10 μM antimycin A, which served as a positive control for complete cell death.

To control for potential osmotic effects of 10 mM β-hydroxybutyrate (BHB), parallel cultures were treated with 10 mM mannitol in the presence or absence of WZB117. Additionally, to determine whether lower, more physiologically relevant concentrations of BHB could produce similar protective effects, separate experiments were conducted with BHB at 1, 2.5, 5, and 10 mM in WZB117-treated cultures, assessing cell viability and mitochondrial membrane potential.

The concentration of BHB (10 mM) was selected based on pilot experiments and previous studies demonstrating neuronal protection with ketone bodies in the range of 5-10 mM in vitro [21]. Subsequent concentration-response experiments (1-10 mM) were performed to establish the minimal effective concentration.

Measurement of mitochondrial membrane potential

Mitochondrial membrane potential was assessed using tetramethylrhodamine ethyl ester (TMRE), a cell-permeant, positively charged fluorescent dye that accumulates in active mitochondria in proportion to membrane potential; thus, decreased fluorescence reflects mitochondrial depolarization and bioenergetic dysfunction. Neurons were seeded at a density of 1 × 10⁶ cells per well in 96-well plates and treated for 48 hours. After treatment, cells were washed twice with phosphate-buffered saline and incubated in serum-free medium containing 100 nM TMRE for 15 minutes at 37°C. Excess dye was removed by gentle washing, and fluorescence was measured at λex 549 nm/λem 575 nm using a multimode plate reader. Data were normalized to protein content and expressed as a percentage of control values. FCCP (carbonyl cyanide-4-(trifluoromethoxy)phenylhydrazone)-treated cells served as positive controls for mitochondrial depolarization. FCCP (10 µM) was used as a positive control for mitochondrial depolarization. FCCP-treated cells exhibited TMRE fluorescence of 18.3 ± 2.1% of control values (data not shown), confirming assay validity.

Measurement of β-secretase (BACE-1) activity

β-secretase (BACE1) activity was quantified using the Human BACE1 ELISA Kit (Catalog #ab231971, Abcam, Cambridge, UK) according to the manufacturer's instructions. Although the ELISA antibodies were raised against human BACE1, sequence alignment indicates substantial conservation in the recognized epitope region, and the treatment-dependent changes observed here support functional detection in chick neuronal lysates (76% in the epitope region). Treated cells were harvested, washed with phosphate-buffered saline, and lysed in tris-EDTA buffer using freeze-thaw cycles. Lysates were supplemented with Triton X-100 and analyzed according to the manufacturer’s protocol. BACE-1 concentrations were calculated from a standard curve and normalized to protein content, expressed as ng BACE-1 per mg protein.

Quantification of Aβ42 levels

Intracellular Aβ42 levels were measured using a sandwich ELISA kit (Catalog #KHB3441, Invitrogen, Thermo Fisher Scientific, Waltham, MA) following the manufacturer's instructions. Cell lysates were used for analysis, and synthetic Aβ42 standards were employed to generate calibration curves. Aβ42 concentrations were normalized to protein content and expressed as pg Aβ42 per mg protein. Both intracellular and secreted Aβ42 levels were measured. For secreted Aβ42, conditioned medium was collected at the end of the treatment period, centrifuged at 1000 × g for 5 minutes to remove debris, and used directly in the ELISA. For intracellular Aβ42, cells were washed three times with phosphate buffered saline (PBS) to remove residual medium-associated Aβ, then lysed as described. To confirm assay specificity, selected samples were preincubated with excess synthetic human Aβ42 peptide (10 µg/mL) to compete for antibody binding; signal reduction > 85% was considered evidence of specific detection. Additionally, parallel cultures were treated with the γ-secretase inhibitor DAPT (10 µM) for 48 h to verify that the detected signal derives from β-amyloid precursor protein (APP) processing.

Protein estimation

Total protein concentration was determined using the BCA protein assay. Cell lysates were prepared using lysis buffer and freeze-thaw cycles. Cell lysates were prepared using radioimmunoprecipitation assay (RIPA) lysis buffer (50 mM Tris-HCl, pH 7.4, 150 mM NaCl, 1% NP-40, 0.5% sodium deoxycholate, 0.1% SDS) supplemented with protease inhibitor cocktail (Catalog #P8340, Merck, India). Lysates were subjected to three freeze-thaw cycles (-80°C to 4°C) and centrifuged at 14,000 × g for 15 minutes at 4°C to remove insoluble material. Protein concentration in supernatants was determined using the BCA assay (Catalog #BCA1, Merck, India) with bovine serum albumin as a standard. Bovine serum albumin standards were used to generate standard curves, and absorbance was measured at 562 nm using a multimode microplate reader.

Statistical analysis

All statistical analyses were performed using GraphPad Prism (version 9.0.0; Dotmatics, Boston, MA). Data are presented as mean ± SEM (standard error of the mean) from independent biological replicates. Data distribution was assessed for normality using the Shapiro-Wilk test before parametric statistical analysis. Given the small sample size typical of primary neuronal culture studies, analysis of variance (ANOVA) results were confirmed by non-parametric Kruskal-Wallis testing, which yielded similar significance patterns.

Comparisons among multiple experimental groups were conducted using one-way ANOVA followed by Tukey’s post-hoc multiple comparison test. No paired comparisons were performed. A p-value < 0.05 was considered statistically significant.

In all experiments, n represents the number of independent biological replicates, defined as neuronal cultures prepared from separate dissections performed on different days (each using three to four pooled embryonic forebrains). For each biological replicate, all experimental conditions were tested in duplicate or triplicate technical wells, and the mean of these technical replicates was used as a single data point for that biological replicate. Data points shown in figures represent these biological replicate means. The tight distribution of data reflects the high reproducibility of the culture system and assays; coefficients of variation across biological replicates were < 15% for all endpoints. Exact numbers of biological replicates (n) for each experiment are indicated in the figure legends and tables.

Effect sizes are reported as partial eta squared (η²) for ANOVA analyses. For pairwise comparisons, 95% confidence intervals for mean differences are provided alongside p-values. All statistical tests were two-tailed, and significance was set at α = 0.05.

## Results

Establishment of primary neuronal culture from embryonic chick brain

**Figure 1:**
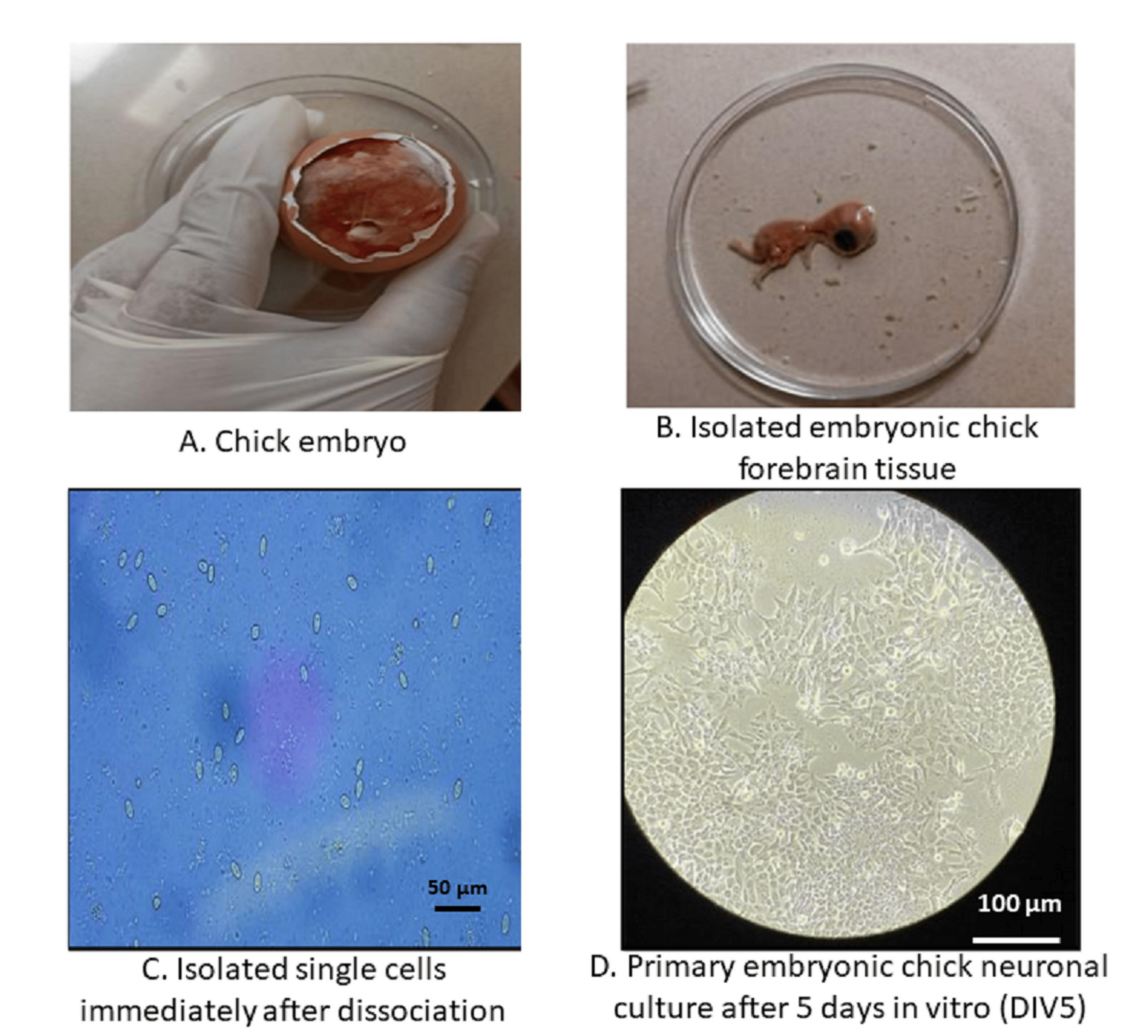
Establishment of primary neuronal culture from chick embryo forebrain (A) Representative image of a chick embryo used for primary neuronal isolation. (B) Isolated embryonic chick forebrain tissue prior to dissociation. (C) Phase-contrast micrograph showing isolated single neurons immediately after dissociation. (D) Primary neuronal culture after five days in vitro, demonstrating extensive neurite outgrowth and network formation.

Primary forebrain neurons were successfully established from embryonic chick brain tissue. Immediately following dissociation, neurons appeared as isolated phase-bright cells with rounded somata and short initial processes. By day 5 in vitro, cultures exhibited extensive neurite outgrowth and formation of interconnected neuronal networks. This morphological maturation confirmed the suitability of the primary neuronal culture system for subsequent metabolic, biochemical, and functional analyses (Figure 1).

Neuronal glucose hypometabolism compromises neuronal viability

The effects of neuronal glucose hypometabolism on neuronal survival were assessed using trypan blue exclusion and lactate dehydrogenase (LDH) release assays after 48 h of treatment. Quantitative outcomes for both assays are summarized in Table 1.

**Table 1:** Quantitative analysis of neuronal viability following neuronal glucose hypometabolism Values represent mean ± SEM from n = 5 independent neuronal culture preparations. One-way ANOVA revealed significant overall treatment effects for trypan blue cell death (F(3,16) = 306.53, p < 0.0001, partial η² = 0.983) and LDH release (F(3,16) = 241.82, p < 0.0001, partial η² = 0.978). Tukey's post-hoc pairwise comparisons: Control vs. WZB117: p < 0.0001 (95% CI for difference: 30.81 to 35.65 for trypan blue; 35.91 to 41.69 for LDH); WZB117 vs. WZB117 + BHB: p < 0.0001 (95% CI: 20.43 to 25.71 for trypan blue; 24.21 to 30.19 for LDH); control vs. BHB: p = 0.892 (95% CI: -3.57 to 1.69 for trypan blue; -4.69 to 2.39 for LDH). * p < 0.001 vs control.​​​ ^†^ p < 0.001 vs WZB117. LDH: Lactate dehydrogenase; SEM: Standard error of the mean; ANOVA: Analysis of variance.

Treatment Group	n	Trypan Blue (%), Mean ± SEM	LDH Release (%), Mean ± SEM	ANOVA F(3,16)	Overall p-Value
Control	5	11.10 ± 1.21	19.8 ± 1.6	306.53	<0.0001
BHB (10 mM)	5	12.04 ± 1.34	11.2 ± 1.9		
WZB117 (60 µM)	5	44.33 ± 0.87*	48.6 ± 2.8*		
WZB117 + BHB	5	21.26 ± 0.79^†^	21.4 ± 2.1^†^		

**Figure 2:**
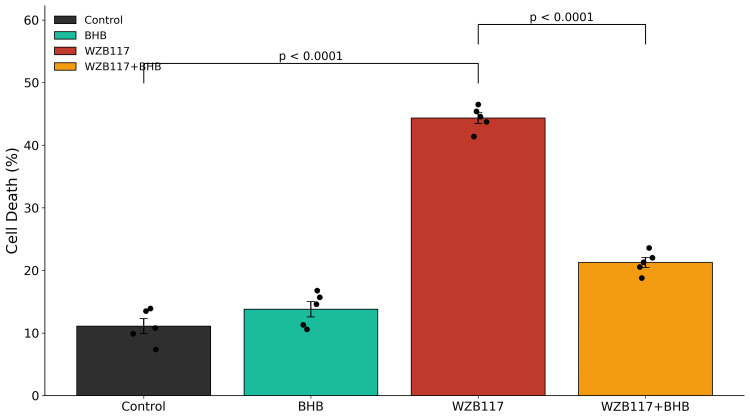
Effects of glucose hypometabolism on neuronal cell death Neuronal cell death was assessed using the trypan blue exclusion assay after 48 h of treatment. Glucose transport inhibition with WZB117 (60 μM) significantly increased neuronal cell death compared with control cultures (44.33 ± 0.87% vs. 11.10 ± 1.21%, p < 0.001), whereas co-treatment with β-hydroxybutyrate (BHB, 10 mM) partially attenuated this effect (21.26 ± 0.79%, p < 0.001 vs. WZB117 alone). Data are presented as mean ± SEM from n = 5 independent neuronal culture preparations. Individual data points represent independent biological replicates. Statistical analysis was performed using one-way ANOVA followed by Tukey's post-hoc multiple comparison test. Significance thresholds: p < 0.001 vs. control; p < 0.001 vs. WZB117. One-way ANOVA demonstrated a significant overall treatment effect (F(3,16) = 306.53, p < 0.0001). Exact ANOVA statistics and the corresponding p-values are reported in Table 1. BHB: β-hydroxybutyrate; ANOVA: Analysis of variance.

Inhibition of glucose transport with WZB117 resulted in a significant increase in neuronal cell death compared with control cultures, as assessed by trypan blue exclusion (Figure 2 and Table 1).

**Figure 3:**
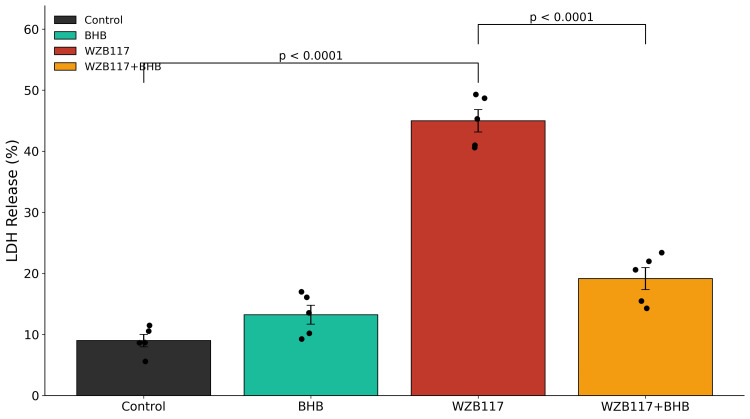
Effects of glucose hypometabolism on LDH release Cytotoxicity was measured by lactate dehydrogenase (LDH) release into the culture medium after 48 h of treatment. WZB117 (60 μM) treatment resulted in a marked increase in LDH release (48.6 ± 2.8%) compared to control cultures (19.8 ± 1.6%, p < 0.001), indicating enhanced neuronal injury. Co-administration of BHB (10 mM) significantly reduced WZB117-induced cytotoxicity (21.4 ± 2.1%, p < 0.001 vs. WZB117 alone). Data are presented as mean ± SEM from n = 5 independent neuronal culture preparations. Individual data points represent independent biological replicates. Statistical analysis was performed using one-way ANOVA followed by Tukey's post-hoc multiple comparison test. Significance thresholds: p < 0.001 vs. control; p < 0.001 vs. WZB117. One-way ANOVA demonstrated a significant overall treatment effect (F(3,16) = 241.82, p < 0.0001). Exact ANOVA statistics and the corresponding p-values are reported in Table 1. BHB: β-hydroxybutyrate; ANOVA: Analysis of variance.

Consistent with this finding, LDH release into the culture medium was also significantly elevated following WZB117 treatment, indicating increased membrane damage and cytotoxicity (Figure 3 and Table 1). Treatment with β-hydroxybutyrate (BHB) alone did not significantly alter neuronal viability relative to control conditions. Notably, co-treatment with BHB significantly attenuated WZB117-induced cytotoxicity in both assays, demonstrating partial preservation of neuronal viability. Full statistical outcomes, including F statistics and exact p-values, are provided in Table 1.

Confirmation of glucose transport inhibition by WZB117

To verify that WZB117 treatment effectively reduced glucose uptake under the experimental conditions, 2-NBDG fluorescence was measured after 24 h of exposure. WZB117 (60 µM) significantly reduced glucose analog uptake to 42.3 ± 4.7% of control values (p < 0.001, n=4), confirming effective inhibition of facilitative glucose transporters. Treatment with WZB117 in medium containing 3-O-methyl-D-glucose, which is transported but not metabolized, did not reproduce the cytotoxic effects observed with glucose deprivation (cell death: 14.2 ± 2.1% vs. 44.33 ± 0.87% in glucose-containing medium; p < 0.001), indicating that the observed phenotypes are attributable to metabolic inhibition rather than nonspecific compound toxicity.

Concentration- and time-dependent effects of WZB117

Preliminary dose-response experiments revealed concentration-dependent effects of WZB117 on neuronal viability and BACE1 activity. Cell death increased progressively from 13.2 ± 1.4% at 10 µM to 44.33 ± 0.87% at 60 µM, with no further increase at 100 µM (46.1 ± 2.3%). Similarly, BACE1 activity increased from 32.4 ± 2.8 ng/mg at 10 µM to 68.9 ± 3.6 ng/mg at 60 µM, plateauing at 100 µM (71.2 ± 4.1 ng/mg). Based on these data, 60 µM was selected for subsequent experiments.

Time-course analysis demonstrated that significant reductions in mitochondrial membrane potential were detectable by 24 h (72.4 ± 3.8% of control, p < 0.01) and became more pronounced at 48 h (59.6 ± 3.9%, p < 0.001) and 72 h (54.2 ± 4.1%, p < 0.001). Cell death increased significantly at 48 h (44.33 ± 0.87%) and 72 h (52.1 ± 2.3%) compared to 24 h (21.3 ± 1.8%, p < 0.01).

Metabolic rescue experiments with β-hydroxybutyrate

To assess whether metabolic supplementation could ameliorate the effects of glucose transport inhibition, parallel cultures were treated with the ketone body β-hydroxybutyrate (BHB). The concentration of BHB (10 mM) was selected based on preliminary experiments and previous studies demonstrating neuronal protection with ketone bodies in the range of 5-10 mM in vitro [21].

To control for potential osmotic effects of 10 mM BHB, parallel cultures were treated with 10 mM mannitol in the presence or absence of WZB117. Additionally, to determine whether lower, more physiologically relevant concentrations of BHB could produce similar protective effects, separate experiments were conducted with BHB at 1, 2.5, 5, and 10 mM in WZB117-treated cultures, assessing cell viability and mitochondrial membrane potential.

All BHB and mannitol treatments were administered concurrently with WZB117 for 48 h, using the same culture conditions described above.

Glucose hypometabolism enhances amyloidogenic processing

To determine whether neuronal glucose hypometabolism influences amyloidogenic pathways, β-secretase (BACE1) activity and intracellular Aβ42 levels were quantified. Quantitative data are presented in Table 2.

**Table 2:** Effects of neuronal glucose hypometabolism on amyloidogenic markers One-way ANOVA revealed significant overall treatment effects for BACE1 activity (F(3,16) = 91.40, p < 0.0001, partial η² = 0.945) and intracellular Aβ42 levels (F(3,16) = 72.63, p < 0.0001, partial η² = 0.932). Tukey's post-hoc pairwise comparisons: Control vs. WZB117: p < 0.0001 (95% CI for BACE1: 34.2 to 46.4 ng/mg; for Aβ42: 98 to 178 pg/mg); WZB117 vs. WZB117 + BHB: p < 0.0001 (95% CI for BACE1: 25.1 to 37.1 ng/mg; for Aβ42: 74 to 158 pg/mg); Control vs. BHB: p = 0.784 (95% CI for BACE1: -7.2 to 9.8 ng/mg; for Aβ42: -42 to 56 pg/mg). * p < 0.001 vs control. ^†^ p < 0.001 vs WZB117. BACE1: β-site amyloid precursor protein-cleaving enzyme 1; Aβ42: Amyloid-β peptide 1–42; ANOVA: Analysis of variance.

Treatment	n	BACE1	Aβ42	ANOVA F(3,16)	Overall p-Value
Control	5	28.6 ± 2.1	214 ± 18	91.4	<0.0001
BHB	5	30.4 ± 2.4	221 ± 21		
WZB117	5	68.9 ± 3.6*	352 ± 29*		
WZB117 + BHB	5	37.8 ± 2.9^†^	236 ± 24^†^		

**Figure 4:**
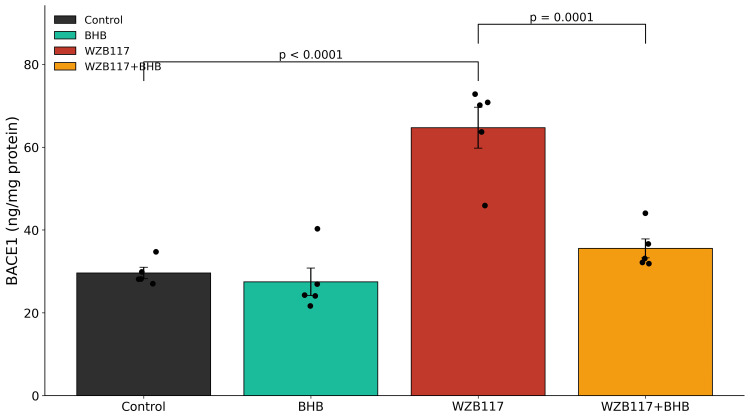
Glucose hypometabolism enhances BACE1 activity β-site amyloid precursor protein-cleaving enzyme-1 (BACE1) activity following 48 h of treatment. Inhibition of glucose transport with WZB117 (60 μM) significantly increased BACE1 activity (68.9 ± 3.6 ng/mg protein) compared with control cultures (28.6 ± 2.1 ng/mg protein, p < 0.001). Co-treatment with BHB (10 mM) partially mitigated this increase (37.8 ± 2.9 ng/mg protein, p < 0.001 vs. WZB117 alone). Data are presented as mean ± SEM from n = 5 independent neuronal culture preparations. Individual data points represent independent biological replicates. Statistical analysis was performed using one-way ANOVA followed by Tukey's post-hoc multiple comparison test. Significance thresholds: p < 0.001 vs. control; p < 0.001 vs. WZB117. Exact ANOVA statistics and the corresponding p-values are reported in Table 2. BACE1: β-site amyloid precursor protein-cleaving enzyme-1; BHB: β-hydroxybutyrate; ANOVA: Analysis of variance.

**Figure 5:**
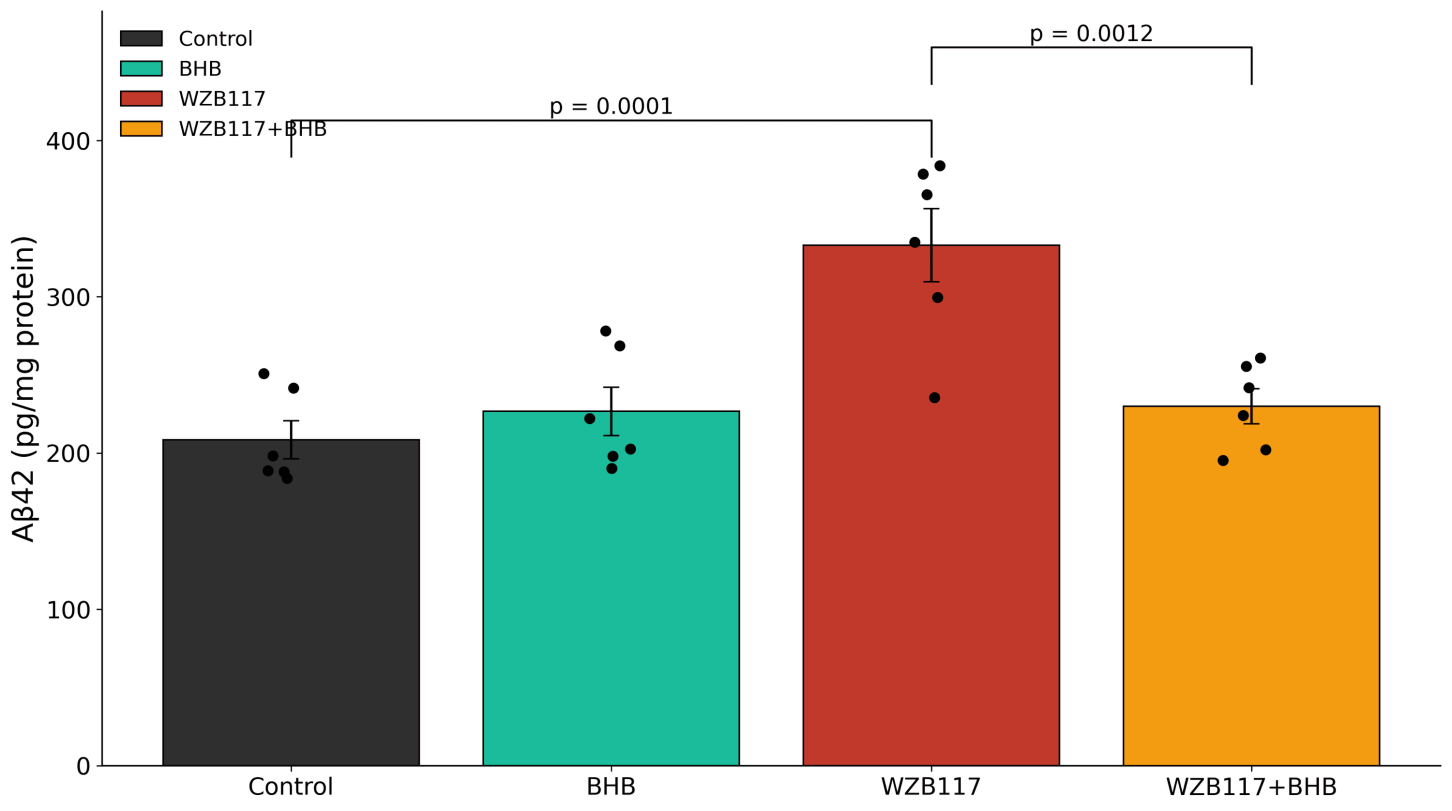
Glucose hypometabolism increases intracellular Aβ42 accumulation Intracellular amyloid-β42 (Aβ42) levels following 48 h of treatment. WZB117 (60 μM) treatment significantly elevated intracellular Aβ42 levels (352 ± 29 pg/mg protein) compared with control cultures (214 ± 18 pg/mg protein, p < 0.001), consistent with enhanced amyloidogenic processing. BHB (10 mM) supplementation significantly reduced Aβ42 accumulation compared with WZB117 treatment alone (236 ± 24 pg/mg protein, p < 0.001). Data are presented as mean ± SEM from n = 5 independent neuronal culture preparations. Individual data points represent independent biological replicates. Statistical analysis was performed using one-way ANOVA followed by Tukey's post-hoc multiple comparison test. Significance thresholds: p < 0.001 vs. control; p < 0.001 vs. WZB117. Exact ANOVA statistics and the corresponding p-values are reported in Table 2. Aβ42: Amyloid-β peptide 1-42; BHB: β-hydroxybutyrate; ANOVA: Analysis of variance.

WZB117 treatment significantly increased BACE1 activity and intracellular Aβ42 accumulation relative to control neurons (Figures 4, 5 and Table 2). BHB treatment alone did not significantly alter either parameter. In contrast, co-treatment with BHB significantly reduced both BACE1 activity and Aβ42 levels compared with WZB117 treatment alone. Complete statistical details are provided in Table 2.

Consistent with enhanced amyloidogenic processing, WZB117 treatment significantly increased intracellular Aβ42 levels from 214 ± 18 pg/mg in controls to 352 ± 29 pg/mg (p < 0.001). Co-treatment with BHB significantly reduced intracellular Aβ42 to 236 ± 24 pg/mg compared to WZB117 alone (p < 0.001).

Treatment with the γ-secretase inhibitor DAPT (10 µM) reduced Aβ42 levels by 78 ± 6% in control cultures and by 72 ± 8% in WZB117-treated cultures, confirming that the measured signal derives from APP processing. Pre-incubation of samples with excess synthetic Aβ42 reduced ELISA signal by 89 ± 4%, supporting the specificity of the assay despite potential species differences.

Specificity of BHB-mediated rescue

To confirm that the protective effects of BHB were attributable to its role as an alternative metabolic substrate rather than osmotic effects, parallel cultures were treated with 10 mM mannitol. Mannitol treatment did not significantly attenuate WZB117-induced cell death (42.8 ± 2.1% vs. 44.33 ± 0.87% with WZB117 alone; p = 0.672) nor restore mitochondrial membrane potential (61.2 ± 4.3% vs. 59.6 ± 3.9%; p = 0.784), indicating that osmotic effects do not account for BHB-mediated protection.

Concentration-response analysis of BHB revealed that protective effects on viability were detectable at 2.5 mM (cell death: 32.4 ± 2.1%, p < 0.05 vs. WZB117 alone), became more pronounced at 5 mM (26.8 ± 1.9%, p < 0.01), and were maximal at 10 mM (21.26 ± 0.79%, p < 0.001). These data demonstrate that BHB exerts concentration-dependent metabolic rescue at concentrations achievable under therapeutic ketosis (2-5 mM), while supraphysiological concentrations (10 mM) produce additional, though modest, benefits.

Glucose hypometabolism disrupts mitochondrial membrane potential

Mitochondrial bioenergetic status was assessed by measuring mitochondrial membrane potential using TMRE fluorescence. Quantitative results are summarized in Table 3.

**Table 3:** Statistical analysis of mitochondrial membrane potential (TMRE fluorescence) following WZB117 treatment Mitochondrial membrane potential was assessed using TMRE fluorescence. One-way ANOVA revealed a significant overall treatment effect (F(3,16) = 84.70, p < 0.0001, partial η² = 0.941). Tukey's post-hoc pairwise comparisons: Control vs. WZB117: p < 0.0001 (95% CI for difference: 32.7 to 48.1%); WZB117 vs. WZB117+BHB: p < 0.0001 (95% CI: 24.3 to 39.9%); control vs. BHB: p = 0.672 (95% CI: -11.2 to 8.6%). * p < 0.001 vs control. ^†^ p < 0.001 vs WZB117. TMRE: Tetramethylrhodamine ethyl ester; ANOVA: Analysis of variance.

Treatment Group	n	TMRE (% of Control), Mean ± SEM	ANOVA F(3,16)	Overall p-Value
Control	5	100.0 ± 4.2	84.7	<0.0001
BHB (10 mM)	5	103.8 ± 5.1		
WZB117 (60 µM)	5	59.6 ± 3.9*		
WZB117 + BHB	5	91.7 ± 4.8^†^		

**Figure 6:**
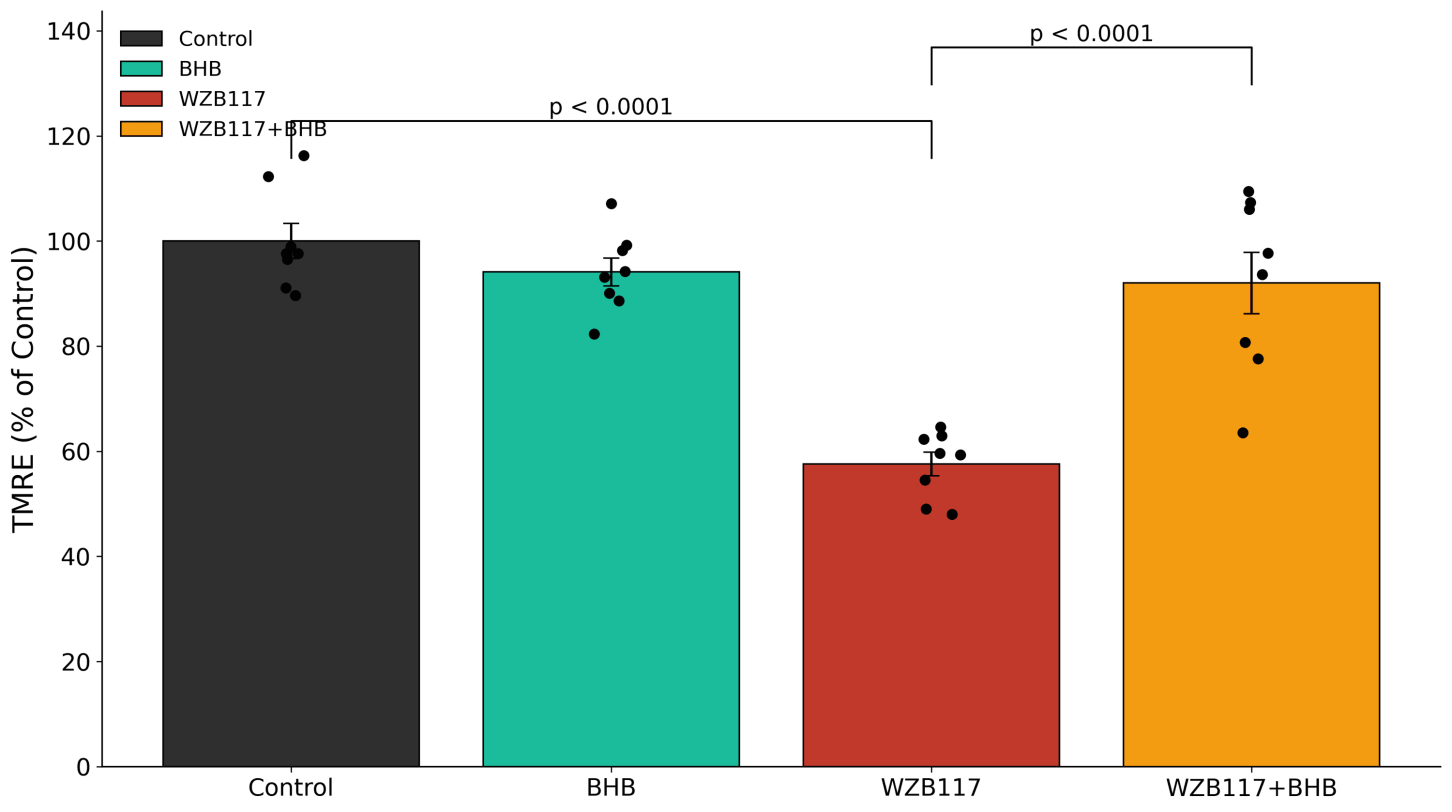
Mitochondrial membrane potential assessed by TMRE fluorescence after 48 h of treatment Mitochondrial membrane potential was assessed using tetramethylrhodamine ethyl ester (TMRE) fluorescence, expressed as a percentage of control. Treatment with WZB117 resulted in a significant reduction in mitochondrial membrane potential, indicating mitochondrial depolarization. Co-treatment with β-hydroxybutyrate partially restored mitochondrial membrane potential compared with WZB117 alone. Data are presented as mean ± SEM from n = 5 independent neuronal culture preparations, with individual points representing biological replicates. Statistical analysis was performed using one-way ANOVA followed by Tukey’s post-hoc multiple comparison test. Significance thresholds: p < 0.001 vs. control; p < 0.001 vs. WZB117. Exact p-values and F statistics are summarized in Table 4. TMRE: Tetramethylrhodamine ethyl ester; BHB: β-hydroxybutyrate; ANOVA: Analysis of variance.

Exposure to WZB117 resulted in a significant reduction in mitochondrial membrane potential compared with control neurons (Figure 6 and Table 3).

BHB alone did not significantly alter TMRE fluorescence. Co-treatment with BHB significantly preserved mitochondrial membrane potential in WZB117-treated neurons. Detailed statistical outcomes are reported in Table 4.

**Table 4:** Descriptive summary of mitochondrial membrane potential (TMRE fluorescence) expressed as percentage of control Values represent mean ± SEM, n = 5 independent experiments. Statistical analysis was performed using one-way analysis of variance (ANOVA), which demonstrated a significant overall treatment effect (F(3,16) = 84.70, p < 0.0001). * p < 0.0001 vs control. ^†^ p < 0.0001 vs WZB117. TMRE: Tetramethylrhodamine ethyl ester; SEM: Standard error of the mean.

Treatment Group	TMRE (% of Control), Mean ± SEM
Control	100 ± 4.8
BHB (10 mM)	97.6 ± 5.1
WZB117 (60 µM)	59.4 ± 4.2*
WZB117 + BHB	92.1 ± 5.0^†^

## Discussion

AD represents the most prevalent neurodegenerative disorder worldwide, with an escalating global burden driven by population aging [1,2]. Despite extensive investigation into amyloid-β (Aβ) and tau pathologies [3-7], disease-modifying therapies remain limited, suggesting that upstream and convergent mechanisms may play a critical role in disease initiation and progression. Among these, cerebral glucose hypometabolism has emerged as one of the earliest and most consistent biological abnormalities observed in AD [8-10]. The present study demonstrates that selective impairment of neuronal glucose utilization induces cellular alterations associated with AD pathology, including increased neuronal vulnerability, mitochondrial depolarization, and enhanced amyloidogenic processing.

Importantly, the model used here isolates neuronal metabolic impairment independent of systemic factors, enabling direct examination of cellular consequences of glucose transport inhibition.

Glucose hypometabolism as a driver of neuronal vulnerability

Functional neuroimaging studies using FDG-PET have repeatedly demonstrated reductions in cerebral glucose uptake in AD-affected brain regions, often preceding overt cognitive impairment and classical neuropathology [8,9]. Importantly, similar metabolic deficits have been observed in individuals at risk for AD, suggesting that glucose hypometabolism is not merely a consequence of neuronal loss but an early pathogenic event [10]. The present findings directly support this interpretation by demonstrating that pharmacological inhibition of neuronal glucose transport alone results in marked cytotoxicity in primary forebrain neurons.

The observed increase in neuronal death following neuronal glucose hypometabolism is consistent with earlier reports of reduced glucose transporter expression at the blood-brain barrier and within cortical neurons in AD brains [11,12]. Neurons depend almost exclusively on glucose-derived ATP under physiological conditions, and sustained impairment of glucose uptake compromises ionic homeostasis, synaptic maintenance, and survival. By reproducing these effects in a controlled in vitro system, the present study isolates glucose hypometabolism as a primary stressor capable of triggering neuronal degeneration.

Mitochondrial dysfunction as a central consequence of energy failure

Mitochondrial dysfunction is increasingly recognized as a central mediator linking metabolic deficits to neurodegeneration in AD [21]. Reduced glucose availability would be expected to limit substrate flux through the tricarboxylic acid cycle and electron transport chain, potentially leading to impaired mitochondrial bioenergetics, consistent with the observed reduction in mitochondrial membrane potential.

These findings are in agreement with previous work demonstrating that mitochondrial abnormalities are present early in AD pathogenesis and may precede amyloid plaque formation [21]. The restoration of mitochondrial membrane potential and cell viability by BHB supplementation, at concentrations as low as 2.5 mM, supports the interpretation that the observed dysfunction is primarily metabolic in origin rather than resulting from nonspecific toxicity. The absence of protection with equimolar mannitol rules out osmotic effects as a confound. These findings are consistent with the established role of ketone bodies as alternative energy substrates that can bypass glycolytic impairments to support mitochondrial respiration [22-24]. Emerging evidence suggests that metabolic stress can directly modulate amyloidogenic processing through transcriptional mechanisms involving NF-κB signaling [25]. This is consistent with previous work showing that energy inhibition elevates β-secretase levels and activity in APP transgenic mice [26]. The concentration-dependent nature of BHB-mediated rescue, with effects detectable at 2.5-5 mM (within the range achieved during therapeutic ketosis), suggests potential translational relevance, though direct extrapolation from in vitro to clinical contexts requires caution.

Emerging evidence suggests that metabolic stress can directly modulate amyloidogenic processing through transcriptional mechanisms involving NF-κB signaling [25].

Integration with the "type 3 diabetes” framework

The concept of AD as “type 3 diabetes” emphasizes brain-specific insulin resistance and glucose utilization defects as central features of disease pathogenesis [15,16]. Insulin signaling regulates neuronal glucose uptake, mitochondrial function, and survival, and disruptions in these pathways have been documented in AD patients [27]. The present study supports this framework by demonstrating that direct impairment of neuronal glucose transport recapitulates multiple hallmarks of AD pathology in the absence of genetic manipulation.

Notably, the protective effects of BHB observed in this study parallel clinical and preclinical findings, suggesting that ketogenic strategies may partially compensate for impaired brain glucose metabolism in aging and AD [22-24]. While the present study does not directly interrogate insulin signaling pathways, the results reinforce the broader view that metabolic interventions targeting neuronal energy failure represent a promising avenue for disease modification.

Metabolic stress and amyloidogenic processing

A key outcome of this study is the demonstration that glucose hypometabolism significantly enhances amyloidogenic processing, as reflected by increased β-secretase (BACE1) activity and elevated intracellular Aβ42 levels. These findings provide mechanistic insights into how metabolic failure may converge with classical amyloid pathology in AD. This is consistent with previous work showing that energy inhibition elevates β-secretase levels and activity in APP transgenic mice [26].

The amyloid hypothesis posits that abnormal production and accumulation of Aβ peptides initiate downstream neurodegenerative cascades [6]. While genetic and proteolytic mechanisms have been extensively studied, accumulating evidence indicates that metabolic stress can directly modulate APP processing. Reduced glucose transporter expression has been shown to correlate with abnormal tau phosphorylation and amyloid pathology in AD brains [13]. Furthermore, impaired glucose metabolism reduces O-GlcNAcylation, a modification that normally suppresses tau hyperphosphorylation and amyloidogenic cleavage of APP [14].

Experimental studies in APP transgenic mouse models have demonstrated that energy inhibition elevates β-secretase levels and activity, thereby enhancing amyloidogenic processing [28]. The present findings are consistent with these observations and extend them by demonstrating that acute neuronal glucose hypometabolism is sufficient to induce amyloidogenic stress in primary neurons. Multiple defects in energy metabolism have been documented in AD, further supporting the link between metabolic dysfunction and neurodegeneration [29]. Importantly, co-treatment with BHB significantly attenuated both BACE1 activity and Aβ42 accumulation, highlighting the dependence of amyloidogenic processing on cellular energy status. While β-hydroxybutyrate was used here as a metabolic rescue agent rather than a therapeutic intervention, these findings support the broader concept that alternative energy substrates can modulate neuronal vulnerability under conditions of glucose restriction.

Experimental model considerations

While chick neuron cultures offer these practical advantages, it is important to acknowledge species differences in APP processing and Aβ sequence. The Aβ42 ELISA utilized in this study detects a chick peptide with structural similarity to human Aβ42, but independent validation with chick-specific reagents would strengthen future studies. Nonetheless, the modulation of signal by WZB117, BHB, and the γ-secretase inhibitor DAPT supports the biological relevance of the measured endpoint. Unlike immortalized cell lines or transgenic models, this system allows direct interrogation of metabolic perturbations without confounding genetic overexpression.

The use of WZB117 to selectively inhibit glucose transport further enables precise modeling of glucose hypometabolism, a feature that is difficult to isolate in whole-animal models where vascular, inflammatory, and systemic metabolic factors interact [17,18]. By combining this model with biochemical and functional endpoints, the present study provides a simplified yet mechanistically informative platform for studying metabolic contributions to AD.

This study has several methodological strengths. First, the use of a primary chick forebrain neuronal culture provides a biologically relevant system that preserves neuronal morphology and metabolic characteristics while allowing controlled experimental manipulation. Second, the study incorporates dose-response and time-course pilot experiments, enabling robust selection of experimental conditions and reducing the likelihood of under- or overestimation of treatment effects. Third, multiple complementary endpoints, including cell viability, mitochondrial function, and amyloidogenic markers, were assessed, strengthening the internal validity of the findings. Finally, the inclusion of metabolic rescue experiments using β-hydroxybutyrate and osmotic controls (mannitol) enhances mechanistic interpretation and minimizes confounding bias.

Limitations

Several limitations should be acknowledged. First, although WZB117 was used to induce neuronal glucose hypometabolism, potential off-target effects cannot be completely excluded; however, the use of metabolic rescue and control conditions supports a predominantly metabolic mechanism. Second, as an in vitro model, this system does not fully recapitulate the cellular complexity of the human brain, including glial interactions, vascular components, and immune responses.

Third, the use of primary chick forebrain neurons introduces potential species-specific differences in amyloid precursor protein processing and metabolic regulation, which may limit direct translational extrapolation to human disease. Fourth, the model reflects acute metabolic inhibition, whereas AD is a chronic, progressive condition; therefore, the temporal dynamics of pathology may differ in vivo.

Fifth, while amyloidogenic and mitochondrial endpoints were comprehensively evaluated, other key pathological features, such as tau phosphorylation, synaptic dysfunction, and neuroinflammatory responses, were not assessed and warrant further investigation. Finally, although ELISA-based detection of Aβ42 demonstrated treatment-dependent changes, the use of antibodies developed against human targets may introduce limitations in sensitivity or specificity in a non-mammalian system.

Future directions

Future studies should extend this model to examine tau pathology, synaptic integrity, and neuroinflammatory signaling under conditions of glucose hypometabolism. Integration with insulin signaling modulators would further clarify the relationship between glucose transport, insulin resistance, and AD pathology. Additionally, longitudinal studies assessing chronic metabolic stress and recovery could provide insights into disease progression and therapeutic windows. The combination of this cellular model with advanced metabolic imaging and omics approaches may further elucidate the complex interplay between energy metabolism and neurodegeneration.

## Conclusions

This study demonstrates that selective impairment of neuronal glucose utilization induces cellular stress responses relevant to AD in a primary forebrain neuronal model. Inhibition of glucose transport resulted in increased neuronal vulnerability, mitochondrial depolarization, and enhanced amyloidogenic processing, indicating that metabolic failure alone can reproduce several cellular features associated with AD pathology. The use of a primary chick neuronal culture enabled direct interrogation of energy-dependent mechanisms in the absence of genetic manipulation or exogenous amyloid exposure, supporting the hypothesis that neuronal glucose hypometabolism may contribute to early pathogenic processes.

Importantly, the partial reversal of neuronal death, mitochondrial dysfunction, and amyloidogenic alterations by β-hydroxybutyrate underscores the specificity and reversibility of metabolic stress and emphasizes neuronal metabolic flexibility. Together, these findings strengthen the concept that disrupted neuronal bioenergetics plays a central role in disease initiation and support metabolism-focused therapeutic strategies. Future studies extending this model to tau pathology, synaptic integrity, and insulin signaling may further elucidate the bioenergetic basis of AD and inform the development of targeted interventions.
